# Effects of continuous prostacyclin infusion on regional blood flow and cerebral vasospasm following subarachnoid haemorrhage: statistical analysis plan for a randomized controlled trial

**DOI:** 10.1186/1745-6215-15-228

**Published:** 2014-06-14

**Authors:** Rune Rasmussen, Marianne Juhler, Jørn Wetterslev

**Affiliations:** 1 Department of Neurosurgery, Copenhagen University Hospital, 9 Blegdamsvej, 2100 Copenhagen, Denmark; 2 Copenhagen Trial Unit, Centre for Clinical Intervention Research, Copenhagen University Hospital, Rigshospitalet 9 Blegdamsvej, 2100 Copenhagen, Denmark

**Keywords:** Subarachnoid haemorrhage, Prostacyclin, Epoprostenol, Vasospasm, Delayed ischaemic neurological deficit

## Abstract

**Background:**

One of the main causes of mortality and morbidity following subarachnoid hemorrhage (SAH) is the development of cerebral vasospasm, a frequent complication arising in the weeks after the initial bleeding. Despite extensive research, no effective treatment of vasospasm exists to date. Prostacyclin is a potent vasodilator and inhibitor of platelet aggregation. *In vitro* models have shown a relaxing effect of prostacyclin after induced contraction in cerebral arteries, and a recent pilot trial showed a positive effect on cerebral vasospasm in a clinical setting. No randomized clinical trials have investigated the possible pharmacodynamic effects of prostacyclin on the human brain following SAH.

**Methods/Design:**

This trial is a single centre, randomized, placebo-controlled, parallel group, double blinded, clinical pilot trial. A total of 90 patients with SAH will be randomized to one of three intervention arms: epoprostenol at 1 ng/kg/min, epoprostenol at 2 ng/kg/min, or placebo in addition to the standard treatment. Trial medication will start on Day 5 after SAH and continue to Day 10. The primary outcome measure is changes in cerebral blood flow measured by a computed tomography (CT) perfusion scan. The secondary outcomes are vasospasm measured by a CT angiography, regional blood flow, clinical symptoms of cerebral ischemia, and outcome at three months (Glasgow Outcome Scale).

**Discussion:**

The primary outcome has been altered slightly since the publication of our study protocol. Global cerebral blood flow is now primary outcome, whereas regional blood flow is a secondary outcome.

**Trial registration:**

Clinicaltrials.gov NCT01447095. Registration date: 11 October 2011.

## Background

Subarachnoid hemorrhage (SAH) accounts for only 5% of strokes but the loss of productive life years due to SAH approaches that for ischaemic stroke and intracerebral haemorrhage [1]. Delayed ischaemic neurological deficit (DIND) is one of the main causes of mortality and morbidity following SAH [2]. Although challenged lately, cerebral vasospasm is still regarded as an important factor in the cause of DIND. Despite extensive research, there is currently no effective treatment for DIND and cerebral vasospasm.

Prostacyclin is an endogenous substance released from the vascular endothelium. It is a potent vasodilator and inhibitor of leukocyte activation, platelet aggregation, and leukocyte-endothelial interactions, which are all potential agents against ischemic event [3]. To date, no randomized clinical trials have investigated the possible effects of prostacyclin on the human brain following SAH.

## Methods/Design

**Figure 1:**
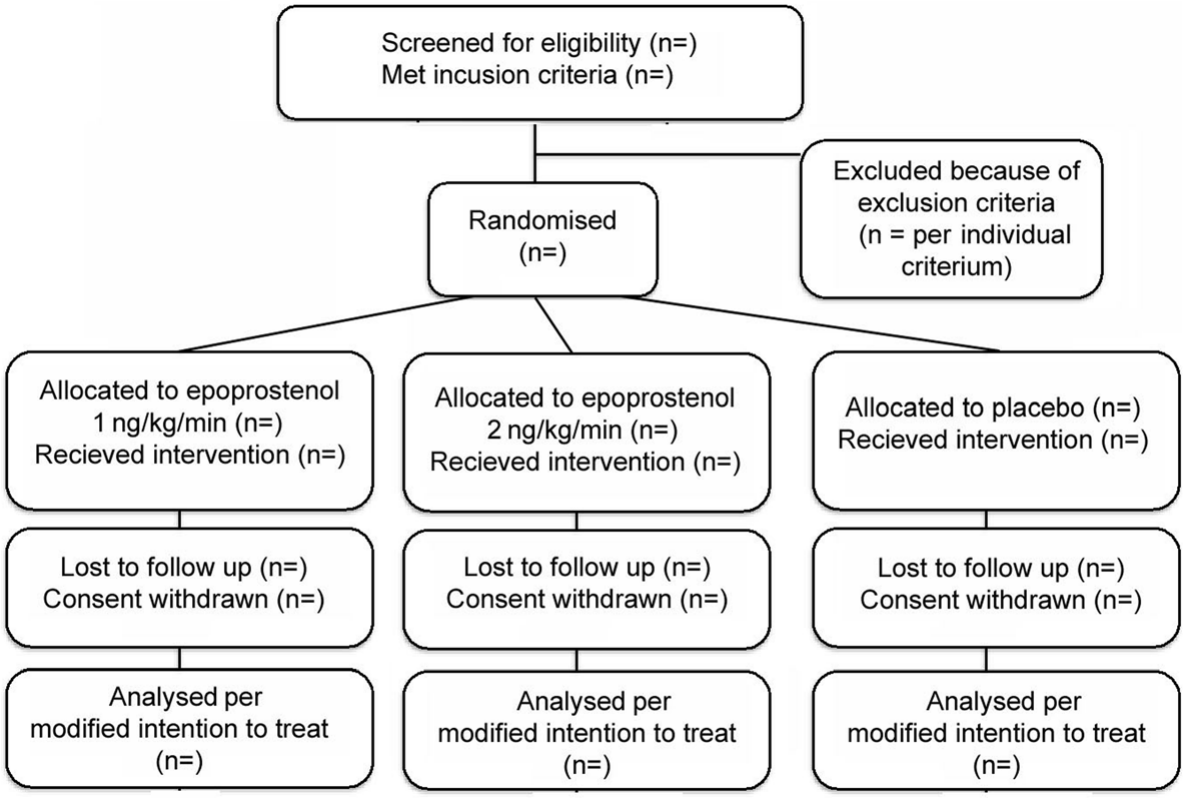
CONSORT flow chart.

This trial is a single centre, randomized, placebo controlled, parallel group, double blindedclinical trial conducted at the Neurointensive Care Unit, of Copenhagen University Hospital. The trial is approved by the Danish ethics committee on human research (reference number H-1-2011-087) and the Danish Medicines Agency (EudraCT 2011-002798–5), and will be carried out in compliance with the Declaration of Helsinki. The trial will be reported in compliance with the CONSORT statement A total of 90 patients will be randomized to one of three intervention arms after informed consent is obtained (see flowchart in Figure 1). No interim analysis will take place. Protocol details are published in the journal *Trials *[4].

### Objective

This trial is an explorative pilot trial designed to investigate the feasibility and possible effects of low-dose prostacyclin on the primary outcome of changes in cerebral blood flow (CBF) from baseline.

The null hypothesis is that there is no difference in changes in CBF from baseline between the three intervention arms (low dose, intermediary dose, and placebo). Based on a type 1 error risk of 5%, a type 2 error risk of 20% (a power of 80%), a standard deviation of 35%, and the possibility to detect or reject a 20% difference between the intervention groups, at least 25 patients per intervention arm will need to be randomized.

## Stratification and design variables

No stratification will take place. Pre-defined design variables allowing for an adjusted analysis of the primary outcome and pre-defined subgroup analyses are: age, sex, Fisher grade, and World Federation of Neurologic Surgeons grade (WFNS).

## Outcome measures

### Primary outcome

The primary outcome is changes from baseline in global CBF, measured by computed tomography perfusion (CT perfusion) in six representative areas in the brain: the arterial territories of the anterior, middle, and posterior cerebral arteries in both hemispheres.

### Secondary outcomes

The secondary outcomes are: 1) occurrence of one or more DIND during the intervention (Day 5 to 10, where ictus is Day 0), 2) cerebral vasospasm qualified as severe, moderate, mild, or absent on a CT angiography, 3) change from baseline in regional blood flow in each of the six vascular territories, and 4) Glasgow Outcome Score (GOS) at three months after SAH.

### Exploratory outcomes

The exploratory outcomes are: the occurrence of elevated transcranial Doppler values (>50% from baseline) in the middle cerebral artery and the number of patients receiving endovascular intervention in the attempt to treat vasospasm.

### Adverse events

The following adverse events will be reported: 1) bleeding (any episode of intracranial, intraspinal, intraocular, intraarticular, pericardial, gastrointestinal, tracheal, oral cavity, nose, or genital bleeding, or bleeding from insertion sites during the intervention period), 2) infection (defined as requiring the administration of antibiotic treatment), and 3) hypotension (>20% drop in mean arterial pressure within one hour after the intervention is initiated).

### Other outcomes: microdialysis and concentration of S100b

Brain microdialysis was planned to be performed on as many study subjects as possible, however, only a small number of patients (12 out of 81 as of March 2014) were monitored with microdialysis due to financial and logistical reasons. The microdialysis data will therefore not be included in the main analyses, but will instead be used in a hypothesis-generating *post hoc* study. Also, the analysis of S100b and other biomarkers will not be reported in the main paper but will be included in the *post hoc* analysis.

## Data points

### Baseline

The baseline variables will be as follows: sex, age, comorbidities (self-reported or reported by relatives) including: hypercholesterolemia, arterial hypertension, previous stroke, diabetes mellitus, and malignancy, and data on admission including: WFNS grade, Fisher grade, aneurism placement, and aneurism treatment mode (clipping or coiling).

### Measurement of outcome variables

A CT perfusion scan will be performed at baseline (Day 2 to 4) and during the intervention (Day 7 to 9). The analysis of the CT perfusion data will be performed by two independent evaluators according to the principles described in the Appendix. The CBF values used in the final analysis will be the average of these two values, however consensus reading will be performed for discordant results (above 20%). A CT angiography will be performed during the intervention (Day 7 to 9).

All patients will be monitored in the ICU and an assessment of neurological performance will be performed every four hours by the nursing staff. On the basis of these observations, the occurrence of DIND as defined by Vergouwen *et al*. [5] will be qualified as present or absent daily by principal investigator. Transcranial Doppler ultrasonography will be performed daily and any occurrence of elevated values (>50 from baseline) will be recorded by principal investigator.

## General analysis principles

All analyses and interpretations of these outcome variables will be performed while preserving the blinding of the statistician and investigators for the interventions. Abstracts presenting the conclusions will be written and approved by the investigators before the blinding is unmasked. Analyses will be conducted according to the modified intention-to-treat principle [6]. All tests of significance will be two-sided with a maximal type-1 error risk of 5%. The primary analyses of primary and secondary outcomes will be those of the modified intention-to-treat population without adjustment for baseline covariates and, if necessary, with data sets generated using multiple imputations.

If there is less than 5% of data missing for a specified primary or secondary outcome we will perform a complete case analysis without imputing the missing values. If there is more than 5% of data missing we will perform Little’s test. If Little’s test indicates that the complete case data set is a random sample we will continue without imputing missing values and analyze the complete cases. If Little’s test indicates that the data set of complete cases is not sampled completely at random of the total data set, we will report the point estimates and their 95% confidence interval applying a worst and best case scenario imputation for the missing values. If the worst and best case analyses allow for the same conclusion we will not perform multiple imputations. However, if the worst and best case imputations provide different conclusions, multiple imputations will be performed, creating 10 imputed data sets under the assumption of data missing at random. The result of the trial will be the pooled intervention effect and 95% CI of the analyses of each data set after multiple imputation.

Multiplicity, a possibly reason for spurious statistically significant *P* values, may be a problem when the result of several outcomes is presented. Addressing this problem is important, but we consider adjustment with Bonferroni correction to be too conservative. For the secondary outcomes we have chosen to consider a *P* value below 0.01 as definitely statistical, whereas a *P* value between 0.01 and 0.05 will be considered indicative of statistical significance. *P* values above 0.05 will be considered as non-significant.

## Statistical analyses

### Trial profile

The flow of study participants will be displayed in a CONSORT diagram as shown in Figure 1 [7]. The number of screened patients who fulfil the study inclusion criteria and the number included in the primary and secondary analyses as well as all reasons for exclusions in the primary and secondary analyses will be reported.

### Primary outcome

CBF is measured by a CT perfusion scan, covering a 4-cm slab of the brain at the level of the basal ganglia. For each patient, six predefined regions of interest (ROIs) will be identified, each being representative of the arterial territory of the anterior, middle, and posterior cerebral artery in both hemispheres.

The continuous outcome of changes in global CBF will be calculated as follows: the CBF value in each ROI is calculated and subtracted from the respective CBF value at baseline and the mean of these six differences is then calculated for each patient. The differences in the means of this value between the three intervention groups will be compared using one-way analysis of variance (ANOVA).

### Secondary outcomes including adverse events

Frequencies and percentages per group will be reported with 95% CI. A standard Chi^2^-test will be used to assess the effect of treatment on binary and categorical outcomes (GOS angiographic vasospasm, increased Doppler values, and occurrence of DIND).

No significance testing will be reported on the individual sub-scores of GOS and the degree of vasospasm. GOS will be dichotomized to grade 1 to 4 (death to moderate disability) and 5 (good outcome). Vasospasm will be dichotomized to none or mild and moderate or severe.

Changes in regional blood flow in each of the six vascular territories will be compared for each intervention group using multiple test of analysis of co-variance (ANCOVA). For adverse events we will perform a Chi^2^-test on patients having one or more adverse events versus patients having no adverse events.

## Outline of figures and tables

Figure one will be a CONSORT flow chart. Figure two will be a bar chart with confidence intervals displaying the mean difference in global cerebral perfusion in the three intervention groups. Figure three will be a bar chart with confidence intervals displaying occurrences of angiographic vasospasm in the three intervention groups.

All tables will report variables according to randomization groups. Table one will report background variables. Table two will report occurrences of DIND. Table three will report occurrences of high velocity Doppler values. Table four will report changes in regional blood flow. Table five will report adverse events. Table six will report endovascular interventions. Table seven will report outcomes for mortality and morbidity at three months with GOS.

## Discussion

The primary outcome described in this statistical plan has been altered slightly compared with the primary outcome described in our study protocol [4]. Originally, we intended to focus exclusively on ROIs with hypoperfusion as these are the most interesting areas regarding the risk of ischemia and development of brain damage. However, a pilot analysis of the CT perfusion data on patients with subarachnoid haemorrhage, performed by our group, has revealed a risk of systematic overestimation of CBF. Other investigators have also addressed problems with precise estimation of absolute CBF values [8,9], although CBF quantification by CT perfusion has been validated [10]. Systematic overestimation of CBF could result in ROIs being discarded as unimportant even if there is a large decrease in CBF from baseline. To allow for an unbiased analysis including all ROIs, and to minimize the impact of possible overestimation, we have decided *a priori* to modify the primary outcome as described above before the database is accessed.

## Trial status

Recruitment started in October 2011 and is currently ongoing.

## Appendix

Analysis of CT perfusion data will be made according to the following principles:

• All analyses of ROIs will be performed on a Philips EBW v. 4.5 PC using the software package ‘brain perfusion’ (Philips Healthcare, P.O. Box 10.000, 5680 DA Best, The Netherlands).

• Mask settings will be <15 HU and >400 HU to exclude bone and cerebrospinal fluid from the analysis.

• Vessels in all ROIs will be removed by the software by checking the ‘remove vessels’ option.

• When selecting the reference artery, the ‘time to peak’ for all main arteries will be calculated. The artery that peaks first will be selected as reference.

• When selecting the reference vein, the vein with the highest net enhancement will be selected.

## Abbreviations

ANCOVA: analysis of co-variance; ANOVA: analysis of variance; CBF: cerebral blood flow; CT: computed tomography; DIND: Delayed ischaemic neurological deficit; GOS: Glasgow Outcome Score; ROI: region of interest; SAH: Subarachnoid haemorrhagehemorrhage; WFNS: World Federation of Neurologic Surgeons.

## Competing interests

The authors declare that they have no competing interests.

## Authors’ contributions

RR and JW proposed the statistical analysis plan. RR drafted the manuscript. MJ critically revised the manuscript for important intellectual content. All authors read and approved the statistical analysis plan and the final manuscript.
